# Huge Intraneural Ganglion Cyst of Tibial Nerve in a 78-Year-Old Male Patient With Gonarthrosis: A Case Report and Review of the Literature

**DOI:** 10.7759/cureus.68740

**Published:** 2024-09-05

**Authors:** Kivanc Yangi, Doga D Demir, Okan Ince, Marion Hof

**Affiliations:** 1 Neurological Surgery, Prof. Dr. Cemil Tascioglu City Hospital, Istanbul, TUR; 2 Emergency Medicine, Prof. Dr. Cemil Tascioglu City Hospital, Istanbul, TUR; 3 Radiology, Karabuk University Training and Research Hospital, Karabuk, TUR; 4 Neurological Surgery, Uniklinik Köln, Cologne, DEU

**Keywords:** gonarthrosis, intraneural ganglia, intraneural ganglion cyst, knee osteoarthritis/koa, tibial nerve

## Abstract

Intraneural ganglion cysts (IGCs) are mucinous cysts located within peripheral nerves, often associated with an articular nerve branch and the adjacent synovial joint capsule. These cysts, while rare, can occur in various nerves, with the tibial nerve being an infrequent site. Tibial nerve IGCs are rare pathologies. We present a case of a tibial nerve IGC in a 78-year-old male patient with pre-existing grade III gonarthrosis. Furthermore, we performed a brief review of the existing literature for tibial nerve IGCs. To our knowledge, we present the second case of an IGC in a patient with known pre-existing gonarthrosis. This case raises the potential association between IGCs and degenerative knee pathologies and underscores the crucial role of early and accurate diagnosis. Differential diagnosis of nerve sheath tumors and extra-articular calf neuropathy is essential not only for definitive treatment but also to rule out more serious alternative diagnoses. While ultrasound-guided aspiration of cystic fluid with steroid injection and conservative management are also treatment methods defined in the literature, we believe that exploratory surgery is the critical point of treatment. Early and accurate diagnosis is paramount, as delayed diagnosis and treatment may cause persistent functional and sensory deficits.

## Introduction

Intraneural ganglion cysts (IGC) are a rare cause of neuropathy and neurologic deficits and have been described in the early 20th century [1]. These benign lesions are usually located close to the synovial joints and are generally mucinous cysts within the peripheral nerves' epineurium. Most commonly, they appear in the peroneal and tibial nerves; however, they can also arise from radial, ulnar, median, sciatic, and posterior interosseous nerves [2-4]. IGCs can also be seen in the pediatric population, although they are more common in adults.

In the knee region, IGCs of the common peroneal nerve are more frequent than the cysts of the tibial nerve. The first IGC of the tibial nerve was described in 1967 [5]. Patients with IGCs are usually admitted to hospitals with symptoms associated with the innervation fields of the involved nerve. Pathophysiological mechanisms should be carefully examined to understand this rare entity's etiology [6,7].

Ultrasound (USG), magnetic resonance imaging (MRI), and electrophysiological studies help diagnose these cysts [8]. The differential diagnosis should include nerve sheath tumors and extra-articular calf neuropathy [9]. USG-guided aspiration of the cystic fluid with steroid injection, conservative management, and exploratory surgery are the treatment methods of IGCs defined in the literature [9,10].

Here we report a 78-year-old male patient with impaired motor function of the left lower leg caused by a vast ganglion cyst of the left tibial nerve and a brief overview of the existing literature.

## Case presentation

The patient, a 78-year-old Western-European male, had a 10-year history of long-term steroid usage due to rheumatoid arthritis. He was admitted to a local district hospital's orthopedics and traumatology department with left knee pain and was diagnosed with grade III gonarthrosis. The patient had no history of trauma or any previous surgery. The patient's family history includes hypertension and diabetes mellitus. He was a non-smoker and did not use any illicit drugs. His rheumatoid arthritis was managed with a combination of disease-modifying antirheumatic drugs (DMARDs) and corticosteroids, with the latter being the cause of his long-term steroid usage. He had been experiencing increasing knee pain and stiffness over the past year, which was not responding well to his current medication regimen.

**Figure 1:**
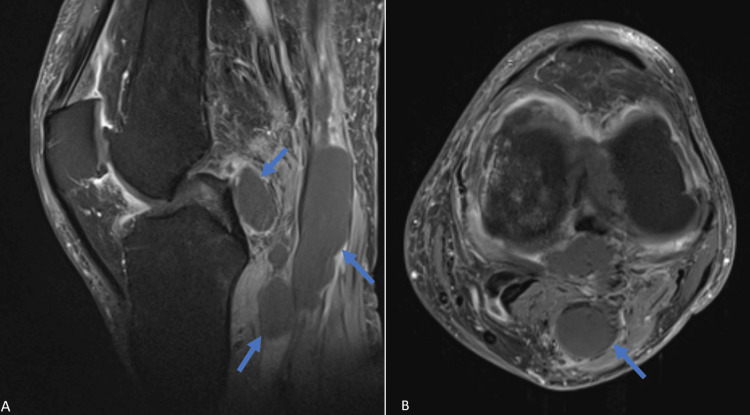
Preoperative T1-weighted contrast-enhanced MRI of the left knee A: Preoperative T1-weighted contrast-enhanced MRI of the left knee, sagittal scan, showing the cystic lesions (blue arrows) with no pathologic contrast-enhancement. B: Preoperative T1-weighted contrast-enhanced MRI of the left knee, axial scan, showing a well-circumscribed cystic lesion (blue arrow) with no pathologic contrast-enhancement.

**Figure 2:**
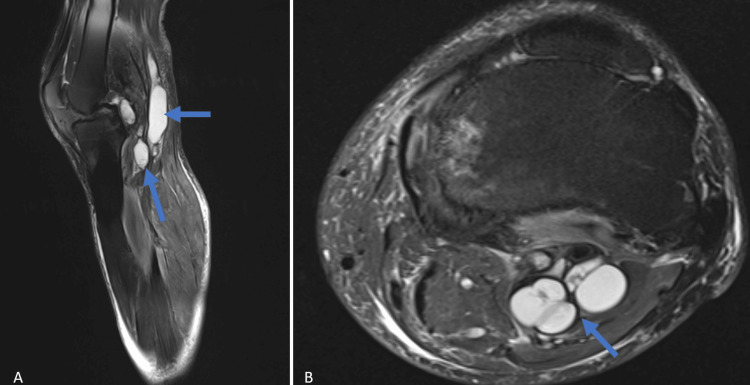
Preoperative fat-saturated T2-weighted MRI of the left knee A: Preoperative fat-saturated T2-weighted MRI of the left knee, sagittal scan, showing multiple hyperintense cystic lesions in the popliteal fossa (blue arrows). B: Preoperative fat-saturated T2-weighted MRI of the left knee, axial scan, showing two well-circumscribed, multilobulated hyperintense cystic lesions (blue arrow).

**Figure 3:**
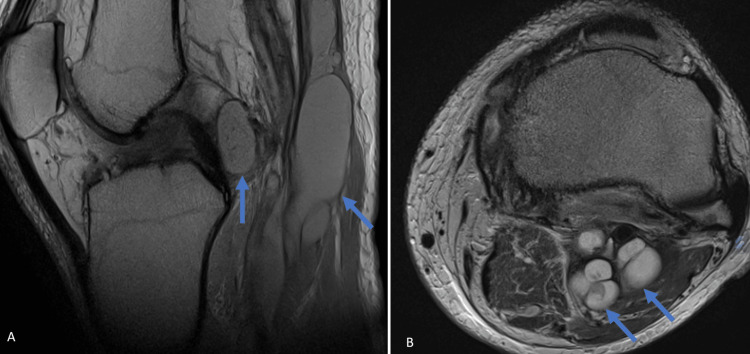
Preoperative T1-weighted non-contrast enhanced MRI of the left knee A: Preoperative T1-weighted non-contrast enhanced MRI of the left knee, sagittal scan, showing multiple iso-hyperintense well-circumscribed cystic lesions (blue arrows) in the popliteal fossa. B: Preoperative T1-weighted non-contrast enhanced MRI of the left knee, axial scan, showing two multilobulated, well-circumscribed hyperintense cystic lesions (blue arrows). Hyperintensity of the lesions is consistent with their mucinous components.

Because of an uncommon swelling of the left lower extremity, an MRI scan of the left knee was performed (Figures 1-3). Under the differential diagnosis of a nerve-sheath tumor, he had consulted for neurosurgery.

The patient's left lower extremity was erythematous and swollen during the physical examination; however, the laboratory investigations were unremarkable. The neurological examination showed an incomplete palsy of the left posterior tibialis muscle, and the toe flexion was impaired (3/5, according to the Manual Muscle Testing Grading System). Sensory deficits could not be found. Furthermore, the patient reported that the swelling suddenly occurred within one day, approximately three weeks before the patient's first admission to neurosurgery. He did not notice any neurological deficits prior to our examination. Electrophysiological findings were consistent with a lesion of the left tibial nerve, and signs of denervation in the left posterior tibialis muscle were found. The patient's pain was unresponsive to the analgesic medications. The surgery was decided to explore the lesion and clarify the etiology of the patient's resistant pain.

**Figure 4:**
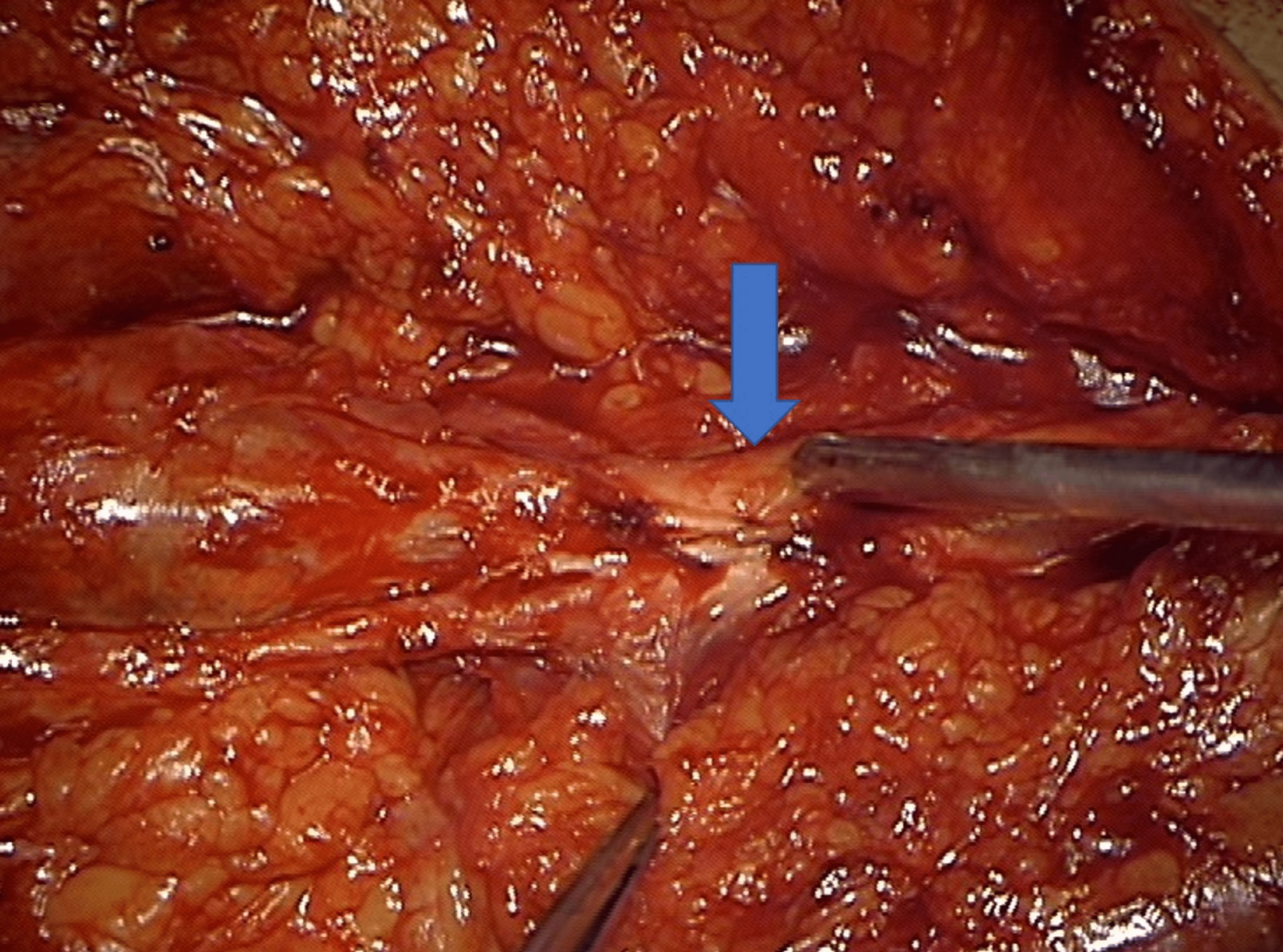
Intraoperative picture: the tibial nerve seemed swollen and thicker than usual (blue arrow)

Intraoperatively, the tibial nerve seemed swollen and thicker than usual (Figure 4). The alteration was started immediately after the terminal division of the sciatic nerve into the tibial and common peroneal nerves, reaching approximately 10 cm below the popliteal area. Corresponding to the MRI scans in the popliteal region, cystic dilatations were found (Figures 1-3). Under electrophysiological control, all of the cystic lesions were explored and drained. The cysts' contents were yellow and mucinous. The fluid was also sent for histopathological examination. Careful examination could not identify tumoral tissue other than extremely edematous neural tissue. Surprisingly, no articular branches associated with the joints could be found. Most noticeable was the alteration of the surrounding connective tissue, where anatomic structures seemed to adhere to each other. An intraoperative frozen section analysis of a small sample of the altered tibial nerve only showed inflammatory changes in the neural tissue. Based on these findings, the operation was terminated. Postoperatively, the patient's left knee pain was completely relieved. On the postoperative first day, the toe flexion was found to be improved (4/5, according to the Manual Muscle Testing Grading System). Histopathological examination results were consistent with a fibrous-walled cyst.

Therefore, a vast IGC of the tibial nerve was diagnosed. Then the patient was transferred to the orthopedics and traumatology department to be further examined for his gonarthrosis. No signs of recurrent symptoms or neurological deficits were found on the patient's first-year follow-up.

## Discussion

A Medline (PubMed) advanced search was performed in April 2023 using the keywords (intraneural ganglion cyst) and (intraneural ganglia). The keywords were linked using the Boolean operator ''OR'' to increase comprehensiveness. The keywords were filtered by abstract and title. About 275 articles were initially retrieved. Only 191 articles, including the case presentations, were selected. Since our study concerns the tibial nerve, 43 articles out of 191, including only the tibial nerve, were selected. Sixteen articles reporting the IGCs of the terminal branches of the tibial nerve were excluded. Repetitive reports due to the cyst recurrence, studies that did not involve case presentations, editorial letters, and old literature reviews that did not involve new case presentations were excluded. Reference lists of the selected studies were also checked. Our literature review revealed that 27 cases of IGCs of the tibial nerve have been reported, excluding the terminal branches, up to the date of our study (Table 1). Moreover, when further data analysis was performed, only one case of an IGC of the tibial nerve in a patient with pre-diagnosed gonarthrosis was found. To our knowledge, this is the second case of an IGC of the tibial nerve in a patient with pre-existing gonarthrosis [1,5,7-30].

**Table 1:** Literature review: detailed analysis of the 27 cases of tibial nerve IGCs ^*^: articles with abstracts or full texts are not available; ^**^: articles that are not available in English; ^a^: patient 1; ^b^: patient 2 IGCs: intraneural ganglion cysts

Reference	Year	Age	Sex	Side	Symptoms	Management	Joint connection
Friedlander et al.^*^ [5]	1967	22	Male	-	Pain	Excision	Absent
Mahaley [11]	1974	20	Male	Left	Left thigh and leg pain	Excision	Absent
Jacobs et al. [12]	1975	21	Male	Left	Numbness and tingling on the left foot	Excision	-
Penkert et al.^**^ [13]	1984	-	-	-	-	-	-
Poppi et al. [14]	1989	61	Male	Left	Left toe paresthesia	Excision	Absent
Spinneret al. [15]	2000	40	Female	Right	Posterior right leg pain	Excision	Proximal tibiofibular joint
Adn et al. [16]	2006	40	Male	Right	Right foot pain	Excision	Absent
Gosk et al.^**^ [17]	2007	-	-	-	-	-	-
Spinner et al. [18]	2009	24	Male	Right	Right leg pain	Excision	Superior tibiofibular joint
Davis et al. [19]	2011	15^a^ 45^b^	2 male patients	Both right	Popliteal pain^a^ Right foot pain^b^	Excision in both cases	Superior tibiofibular joint^a^ Subtalar joint^b^
Jose et al. [10]	2011	36	Male	Right	Posterior knee pain	US-guided aspiration and steroid injection	Proximal tibiofibular joint
Spinner et al. [1]	2011	46	Male	Left	Proximal leg pain	Excision	Superior tibiofibular joint
Squires et al. [7]	2014	10	Male	Left	Intermittent posterior left knee pain	Unsuccessful US-guided aspiration followed by surgical excision	Superior tibiofibular joint
Jerath et al. [20]	2014	59	Male	Left	left posterior thigh pain and complete motor paralysis of the foot.	Excision	Tibial articular branch of the sciatic nerve (joint name was not mentioned)
Palit et al. [21]	2015	16	Male	Right	Right popliteal fossa discomfort	Excision	Proximal tibiofibular joint
Isaacs et al. [22]	2016	24	Male	Right	Right foot and ankle pain	Excision	Subtalar joint
Silveiraet al. [9]	2017	51	Male	Left	Left ankle paresthesia	Conservative	Absent
Buckley et al. [23]	2017	39	Male	Left	Foot pain, dorsiflexion weakness	Excision	Suspected proximal tibiofibular joint connection
Migonis et al. [24]	2019	56	Male	Left	Tarsal tunnel syndrome	Excision	Medial subtalar joint
Igielska-Bela et al. [25]	2019	37	Male	Right	Right foot pain and paresthesia	Excision	Absent
Pufferet al. [26]	2019	52	Male	-	Medial plantar paresthesia	Unsuccessful US-guided aspiration followed by surgical excision	Subtalar joint
Wu et al. [8]	2019	49	Male	Left	Medial ankle numbness	Excision	Absent
Eguchi et al.^**^ [27]	2020	39	Male	Right	Right toe dorsiflexion difficulty	Conservative	-
Mayer et al. [28]	2021	61	Male	Left	Foot drop, medial-left hamstring pain	Excision	Superior tibiofibular joint
Wang et al. [29]	2021	64	Female	Right	Right calf pain	Excision	Superior tibiofibular joint
Vetchy et al.^*^ [30]	2023	56	Male	Right	Dysesthesia of posterior lower limb	-	Superior femoral joint capsule branch of the posterior knee joint

IGCs are rare but benign lesions of the peripheral nerves. These lesions can easily be confused with schwannomas and other peripheral nerve sheath tumors since their clinical and radiological features resemble tumoral and inflammatory reactions. Differentiation between extraneural (e.g., Baker cysts) and intraneural cysts is also essential and based on the joint connections via an articular branch. This differentiation can be best done with MRI scans [31]. Although conservative management and USG-guided aspiration are also included in the management of these cysts, if an articular branch is observed on MRI, the treatment of choice should be exploratory surgery.

Three theories - the degenerative, unifying articular, and tumoral-based theories - are trying to explain IGCs' origin and mechanical evolution. The unifying articular theory is the most recent and widely accepted. According to this theory, the cyst originates from synovial fluid that leaks through capsular defects of the synovial joints and proceeds from the least resistant area: cystic fluid gets outside the degenerated synovial joint via the articular nerve branch. Propagation of the cyst follows the nerve sheath of the parent nerve and other major nerves, terminal nerve branches, and even nearby located vessels. Intraneural and extraneural pressure fluxes affect the cyst's propagation, final size, and dimension [6,28]. MR imaging may show a characteristic “tail sign,” representing the connection of the joint and the intraneural cyst [4].

Furthermore, recent studies in the literature have demonstrated that IGCs can also occur without the existence of the articular branch [5,8,9,11,14,25]. In our case, an articular branch associated with the adjacent joints was not observed on MRI scans and could not be found macroscopically during the operation. If there is a connecting articular branch, surgical exploration should be considered, and this branch must be disconnected from the relevant joint to relieve the patient's symptoms. It has been reported that patients' symptoms persist if the articular branch of the involved nerve is not disconnected from the associated joint capsule [4,6]. On the other hand, If the articular branch is exposed but not disconnected from the associated joint, these lesions tend to recur [15].

The etiology of IGCs also includes trauma. As reported in the current literature, these benign mucinous cysts may also occur secondary to trauma [7]; however, in our case, the patient had no history of trauma. Our literature review has also shown that, in patients with tibial nerve IGCs, the tibiofibular joint is the most commonly associated joint with the articular branches of the tibial nerve, followed by the subtalar joint [1,7,10,15,18,19,21,23,28].

Patients with IGCs are usually present with pain, paresthesias, or motor impairments. Pain in the knee region is generally associated with degenerative changes in the knee joint but can also be related to degenerative meniscal tears or Baker's cysts [32]. In our case, the patient was diagnosed with grade III gonarthrosis. Can gonarthrosis increase the tendency for these cysts to occur? A recent study has shown that degenerative joint pathologies such as knee osteoarthritis cause inflammation in the synovial membrane of the joints [33]. Inflammation of the synovial membrane is highly associated with pain. Chronic degenerative joint pathologies may have an essential role in the etiology of the IGCs, as previously stated in the literature; however, to state that there is an exact causative relationship between these cysts and gonarthrosis, further studies focus on the pathophysiologic mechanisms are needed.

## Conclusions

IGCs are benign lesions of the peripheral nerves. Despite their benign nature, they may cause crucial problems, such as paresthesias and motor weaknesses. IGCs of the tibial nerve are rare and generally associated with an articular branch propagating into the tibiofibular or subtalar joints. In our case, there was no history of trauma and no articular branch associated with the adjacent joints, suggesting that the etiology of IGCs is multifactorial. Gonarthrosis may be a facilitating factor for the occurrence of these cysts; however, further studies involving more patients are needed to enlighten the relationship between the IGCs and degenerative knee pathologies. According to our knowledge, early and accurate diagnosis and exploratory surgery are essential in the management of IGCs since delayed diagnosis may cause persistent symptoms and further neurological deficits.
